# Supplementary figures and images for: Adaptive oscillators support Bayesian prediction in temporal processing
Source: PLoS Comput Biol. 2023 Nov 27;19(11):e1011669. doi: 10.1371/journal.pcbi.1011669 (PMC10703266; doi:10.1371/journal.pcbi.1011669)

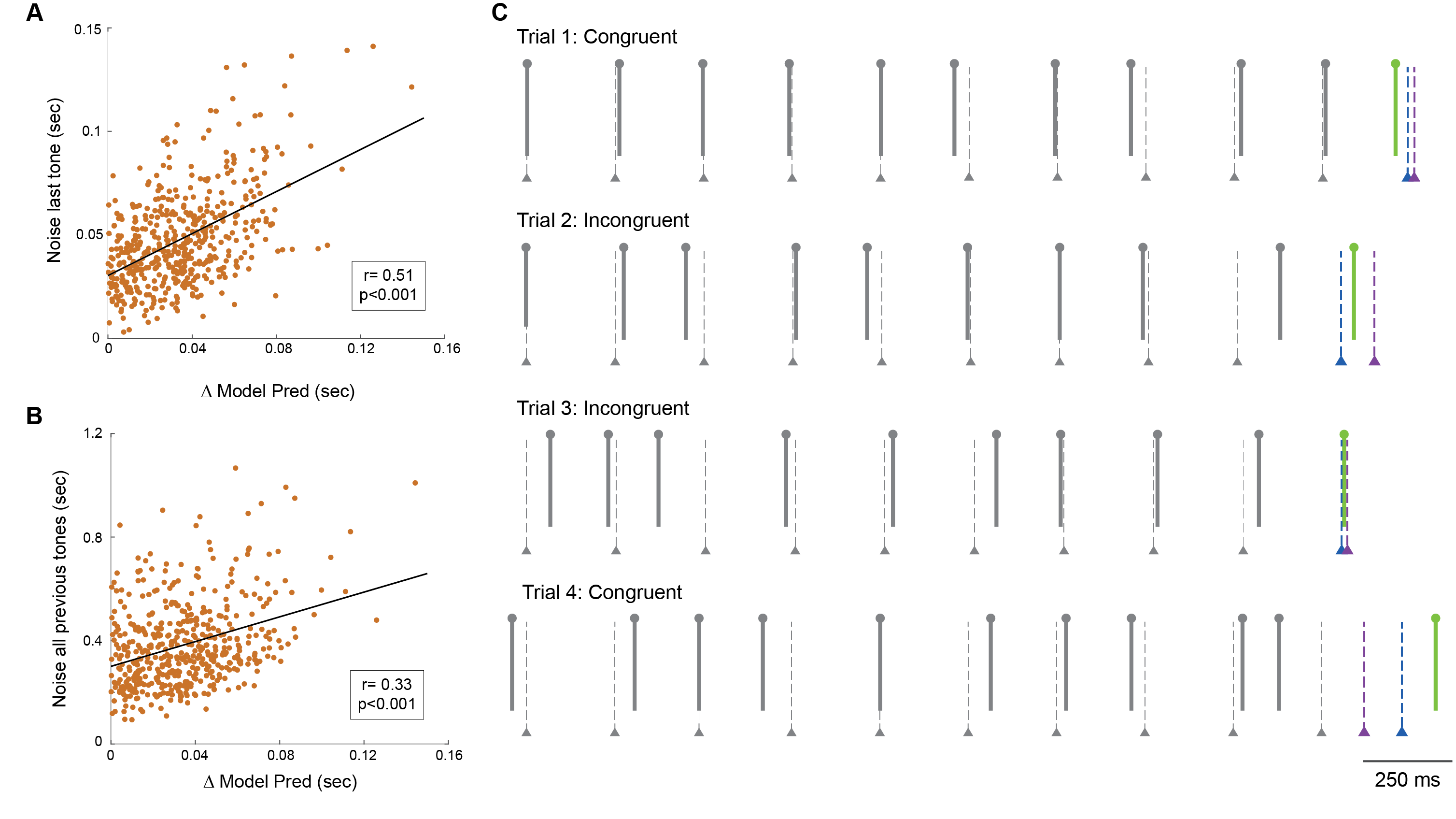

Supplement: S1 Fig — A. Relationship between the amount of noise in the last tone of the sequence (how much it departs from the underlying rhythmic structure) and the difference between the probe’s time predicted by the two models. B. Relationship between the sum of the noises in the sequence of tones preceding the last one and the difference between the probe’s time predicted by the two models. Panels A&B imply that the more the trials depart from the perfect isochronous case, the larger the difference between the models predictions. Additionally, accordingly to how the jitter accumulates on the different models, the noise in the last tone has the strongest effect in differentiating both predictions. Each dot represents one trial and the solid line a linear regression of the data. C. Schematic representation of four trials of the 4Hz rhythm condition. Dashed gray lines: Underlying rhythmic structure (i.e., where the tones of the sequence should be presented for a perfect isochronous stimulus). Solid gray lines: Presented tones. Green: Probe tone. Dashed blue and magenta line: Probe’s predicted time according to the relative and absolute perceptual timing, respectively. Congruent trial: both models agree on the probe being early or late. Incongruent trial: there is a disagreement between the models categorical outcome (i.e., probe happens early or late). As exemplified by this figure, the different models’ categorical outcome not only depend on the trail structure but also on the timing of the probe tone. While it’s true that the larger the difference between the models predictions the more likely is to get an incongruent trial and vice versa (i.e., Trials 1 and 2), it’s also possible to find trials with: 1. a small difference between models but with the probe taking place in the middle granting an incongruent trial (i.e., early or late, Trial 3); or 2. a large difference between models but both lying on the same side of the probe granting a congruent trial (i.e., early or late, Trial 4 [file pcbi.1011669.s001.png]

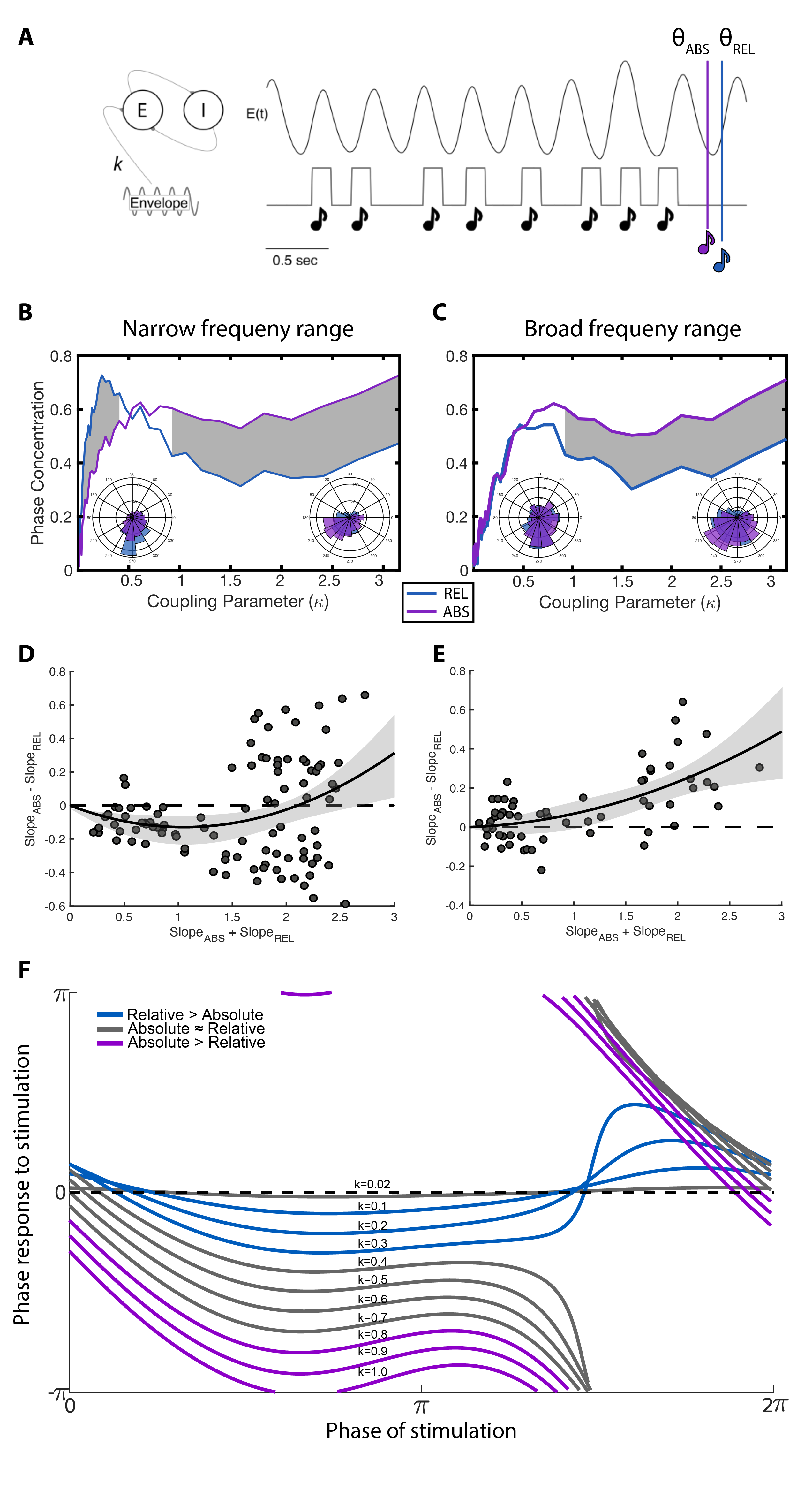

Supplement: S2 Fig — A. Set up of a Wilson-Cowan oscillator model (see Methods) with parameters set at: a = b = c = 10, d = -2 and (E,I) = (1.6, -2.9). The acoustic envelope of a stimulus trial drives the excitatory population with coupling determined by parameter k. The phase of the oscillator at the expected time of the last tone according to absolute timing (ABS) or the relative timing algorithm (REL) is computed on each trial. B. Phase concentration of predicted phases, ABS in purple and REL in blue across trials at a restricted range of stimulus rates (240 to 260 ms). Better phase concentration would lead to a more accurate prediction of the probe time relative to the corresponding perceptual timing mechanism. Shaded areas mark significant differences using the circular K test to test for significant differences in concentration (correcting for multiple comparisons using the false discovery rate Benjamini & Hochberg, 1995). Insets represent example concentrations at = 0.15, left, and = 2.0, right. C. Same as b but with a range of stimulus rates that reflects the statistics of the experiment (210 to 290 ms). D. Model task performance compared between Absolute and Relative algorithms in the restricted range of stimulus rates (240 to 260 ms). The difference in slope parameters fitting a Logistic regression between phase of the oscillator at probe time and the correct response defined either by absolute or relative perceptual timing mechanisms (see Methods). Polynomial fit and confidence shown in black line and gray patch respectively. Second order determined through AIC Model selection. E. Same as D with the broader range of stimulus rates (210 to 290 ms). F. Phase Response Curve of the Wilson-Cowan oscillator in response to a single 100 ms tone at a range of coupling parameters (from 0.02 to 1). Blue lines refer to coupling constants that lead to significantly higher concentration for the relative prediction, Purple to those significantly higher for the absolute one and gray to those [file pcbi.1011669.s002.png]

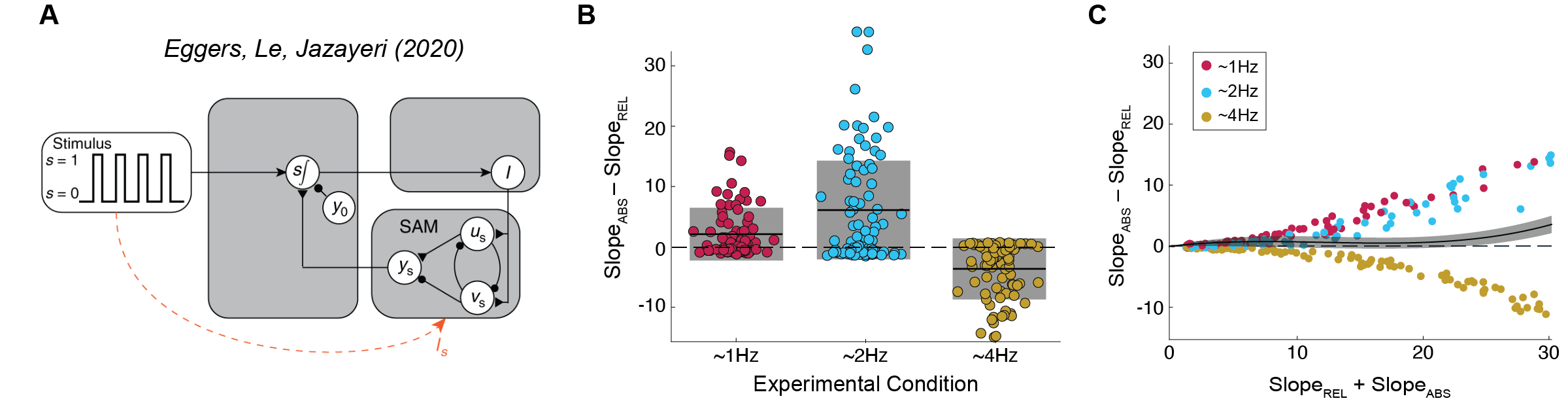

Supplement: S3 Fig — A. Model schematic of the ramping model (adapted from Egger and colleagues23). The model contains two competing units us and vs whose values decay to a stable point, driven by current I controlling the speed of this decay. Their difference yields ys, a ramping value which is compared with threshold, y0, at the time of a stimulus. The difference d = ys—y0 at the time of each tone is used to adjust I controlling the speed of the ramp to reduce d on the next interval. In our case, d is also used at the time of the probe tone to output a response to the behavioral trial. If d > 0, the model responds “late”; if d<0, the model responds “early”. For code and further description of the module, see Eggers et al, 2020. B. The model responses are then treated as behavioral data. Late and early responses are coded as 1 and 0 respectively and a logistic function is fitted, and the slope extracted to identify the precision of the responses relative to the relative and duration algorithms. Same procedure as the one applied to the behavioral data. Slope differences are shown here by stimulus rates at 1.2 Hz (red), 2 Hz (blue) and 4 Hz (gold). C. Slope difference relative to overall performance (slope sum) for the same stimulus rates. Black line represents polynomial fit, while the gray area represents the 95% confidence interval of the mean. Third order polynomial selected through AIC model selection. Data for S3 Fig can be found in S8 Data. (PNG) [file pcbi.1011669.s003.png]

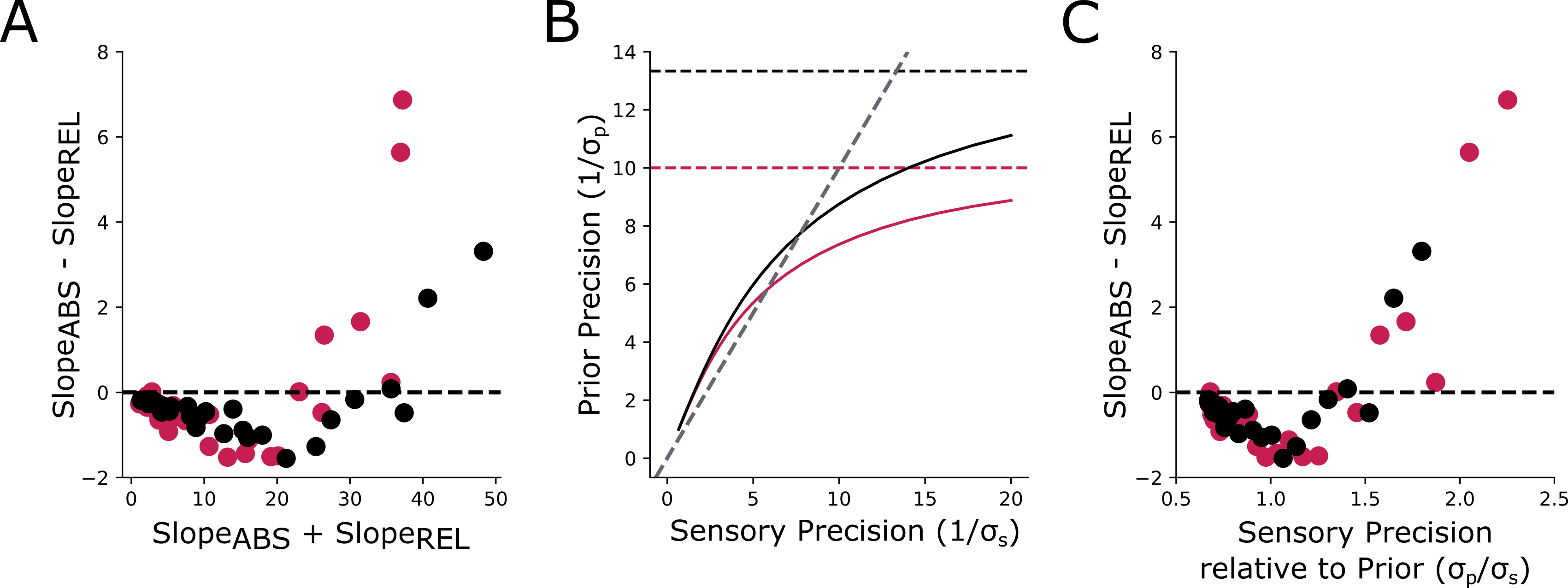

Supplement: S4 Fig — A. Bayesian simulation of the low jitter (black) and high jitter (magenta) conditions. Equivalent to Fig 4F. B. Comparison of the Sensory Precision 1σs with the Prior Precision 1σp in the low jitter (black, solid line) and high jitter (magenta, solid line) conditions. Horizontal dashed lines refer to 1σ for each experimental jitter: σ = .1 for high jitter (magenta) and σ = .075 for low jitter (black). These are the limit of possible precision of the prior if it were 100% confident where the expected tone location would be (considering the unpredictable variance of the experiment). Gray line dashed line refers to the identity line. C. The low and high jitter conditions are fully aligned when explained by sensory precision relative to the prior σpσs. Data for S4 Fig can be found in S9 Data. (PNG) [file pcbi.1011669.s004.png]
