# Supplementary material for: Adaptive oscillators support Bayesian prediction in temporal processing
Source: PLoS Comput Biol. 2023 Nov 27;19(11):e1011669. doi: 10.1371/journal.pcbi.1011669 (PMC10703266; doi:10.1371/journal.pcbi.1011669)
Supplement: Attachment — Submitted filename: Doelling_Arnal_Assaneo_2023_review_v1.pdf [file pcbi.1011669.s014.pdf]

This paper attempts to clarify and reconcile two proposed algorithms for rhythmic timing in humans: absolute timing (in which intervals between events are assumed to be noisy draws from some peaked probability distribution, such as normal) and relative timing (in which the deviations from an assumed underlying rhythm are assumed to be, e.g., normally distributed). In particular, the authors explore the differences between these two timing mechanisms for *irregular* rhythmic stimuli. To do this, the authors use a task in which the goal is to determine whether a "probe tone" is early or late relative to a preceding (quasi-)rhythmic tone sequence. For human subjects, they find that whether subjects seem to rely on absolute or relative timing mechanisms depends on the noisiness of the quasi-rhythmic tone sequence, with absolute and relative mechanisms being preferred at low and high levels of noise, respectively. They show that a similar profile of responses is generated by a modified Wilson-Cowan oscillator which exhibits frequency adaptation, and, interestingly, also by a Bayesian algorithm in which the prior for the timing of the next tone is determined via absolute timing. This paper makes a number of novel and important contributions which potentially bring precision & clarity to the question of absolute vs. relative timing – e.g., exploring rhythmic sequences which exhibit noise or jitter in the timing of successive tones, constructing a version of the Wilson-Cowan oscillator which exhibits frequency adaptation, and demonstrating a preference for relative and absolute timing mechanisms that depends on the level of sensory noise.

However, this reviewer feels the paper could go further to clarify the central questions – the relationship between absolute and relative timing and their equivalence, and the relationships between the various models discussed. This would require changes to the exposition (mostly additions) to further elucidate exactly what is happening and exactly what it is that the authors have found. It would be helpful to introduce some terminology to further clarify the differences between the various uses of absolute and relative timing in the manuscript (see A below) and the structure of the Bayesian model (see C below). As far as terminology goes, I have made some suggestions, but anything sufficiently clear, consistent, and appropriate will work. Two possible further analyses would also be useful in this reviewer's opinion. The first would be to explore which tone sequences lead to different predictions for the absolute and relative timing mechanisms (see B.2 below). The second would be to explore how instantiation of the absolute vs. relative timing mechanism (by the Bayesian model with the prior coming from the absolute timing model) depends on the relative precision of the prior (see C.4 below). I believe these two analyses would greatly improve the manuscript, and it is my guess that they are not too difficult to compute, given the existing analyses, with the second probably easier to execute than the first.

Some major points are as follows:

A. As it stands, there is some confusion as to the various uses of absolute and relative timing throughout the paper.
    1. The terms "mechanism" and "algorithm" are used with absolute vs. relative timing throughout, but I think the exposition would be helped by further distinguishing at least three different uses of absolute and relative timing in the manuscript. First, absolute and relative timing are two different statistical models of irregular or quasi-regular sequences of events, that lead to different statistics for the intervals between events (see B.3 below).

Then, there are the algorithms that the authors introduce to *predict* the timing of an upcoming event in a quasi-rhythmic sequence based on past events. These are inspired by the absolute and relative timing models, but they are not equivalent. One attempts to impute a period T that minimizes the difference between the observed tone time (t_i) and the tone time expected in the absence of noise (i*T); this corresponds to the relative timing model because it assumes normally distributed differences t_i – i*T. The other attempts to find the most likely inter-event-interval t_{i+1} – t_i, assuming that it will be close to the mean of previous inter-event-intervals; this corresponds to the absolute timing model because it assumes normally distributed inter-event-intervals.

Finally, there is the use of these algorithms to produce priors on unobserved tone times, within a Bayesian framework for tone prediction. This Bayesian framework could also be thought of as an "algorithm", but it would be helpful to distinguish it from the algorithms described above, perhaps by referring to it exclusively as a "framework" or "Bayesian model" – for example, the "absolute Bayesian model" and the "relative Bayesian model".

In any case, I think it would be very helpful to outline these distinctions early in the manuscript, perhaps at the beginning of the results section, or perhaps in a "notation" section prior to the results section. It would also be helpful to take pains to specify *which* of these three uses of absolute and relative are intended throughout the manuscript.

2. All three models (human, rhythmic, and Bayesian) are described as showing a "preference" for absolute vs. relative timing, a phrase which I found confusing. In fact, what seems to be happening is that the decisions produced by these systems agree more with the timing determined by regular or absolute timing. I believe terms that reflect this, like "agree with" or "align with" or even "instantiate", would be easier to understand than "prefer". In cases where you're talking about preferential alignment, maybe that phrase could be used.

B. The authors ought to include more explanation about the differences between absolute vs. relative timing. There are several places and ways I believe this could be done.

1. The authors should make clear the statistical models/assumptions underlying absolute and relative timing. E.g., unless I'm mistaken, relative timing assumes that t_i = i*T + \eps_i, while absolute timing assumes t_i – t_{i-1} = T + \eps_i, or that t_i = \sum_1^i(T + \eps_i) = i*T + \sum_i\eps_i. Thus, given that noise is assumed to be drawn from the same distribution for both models, the resulting "prediction error" t_i – i*T is differently distributed. E.g., for gaussian \eps_i, it is assumed to be white noise for relative timing, and brown noise for absolute timing. Thus, as the authors do state in the Methods on page 13, the absolute timing model exhibits a greater probability of drift in the time series. (Other parts of the discussion on page 13 could be brought into the main manuscript, in a section introducing the three uses of absolute & relative.)

Along similar lines, it should be clarified that the time series used in the current experiments are generated from the relative timing model – i.e., they assume a rhythmic event timing T*i and add a gaussian noise to it. This is fine given that the manuscript aims at understanding how humans make *rhythmic* temporal predictions – but it should be mentioned. And, the question of whether humans use different mechanisms

to estimate the timing of noise that is generated via the absolute timing model could be entertained, although in this reviewers opinion this is unlikely (since the manuscript seems to suggest that humans use an absolute timing-inspired algorithm in either case).

2. It should be explained when and how the absolute and relative timing algorithms give different predictions for the timing of the next tone in a sequence. As far as I can tell, the biggest difference here should be that the absolute timing algorithm takes the previous (perceived) tone time at "face value", adding the average inter-tone-interval to it, while the relative timing algorithm assumes that the previous (perceived) tone time is a noisy observation of some expected rhythmic tone timing, and adjusts the frequency and phase of this underlying/expected rhythm to minimize the sum of the squared differences between t_i and i*T. In other words, for the relative timing algorithm, while some of the noise in tone timing results in a different estimate for T, some it can be "absorbed" by shifting the inferred offsets \eps_i of the "observed" tones from the "beat". For the absolute timing algorithm, noise in tone timing always contributes directly to changes in the estimated mean and uncertainty of the inter-tone-interval T. In any case, this issue should be clearly unpacked earlier in the manuscript (the current manuscript has something in this direction in the Methods, on page 13).

   I would also suggest the authors illustrate one or several tone sequences for which the two algorithms give different predictions. They could replace, e.g., the cartoon example in Fig. 1A (in which actual intervals do not appear to be used) with an actual example where the two algorithms differ in their predictions. To go further, they could isolate the trials in which the two algorithms give very different predictions (e.g., the highest quartile in terms of difference, or predictions that are early for one and late for the other), and see what generalizations can be made about the statistics of the noise on those trials. If the reasoning above is correct, one of the largest contributions here should be the amount of noise in the tone before the probe. An illustrative (accurate, cartoon) example would thus be one in which all tones are exactly rhythmic except the one prior to the probe. The magnitude of the noise in this tone should affect prediction more in the absolute timing algorithm. It's possible that a sequence in which the inter-tone-intervals are strictly decreasing or increasing would also yield a large difference in the predictions.

3. The relationship between Bayesian predictions made using relative and absolute timing – i.e., why it is that the Bayesian framework with the absolute timing prior "looks like" the Bayesian framework with the relative timing prior for high levels of noise – ought to be further explained. The closest the authors come to this is the sentence *"When participants are more uncertain about their own sensory measurements, the expectation of similarity between intervals yields a stronger effect of rhythmicity in the estimation."* This could be stated much earlier, and the authors could elaborate and clarify the mechanism through which this occurs. I.e., having explained the differences between the two models and algorithms as suggested above, they could state that the precision of the likelihood of each tone plays a crucial role in determining how close that tone's posterior distribution (and "perceived" timing) is to the prior distribution (which assumes a constant inter-tone-interval, i.e., rhythmicity, for both absolute and rhythmic timing algorithms). Intuitively, when the precision of the observed timing is low, the

posterior distribution acts to "adjust" the location of each tone in order to bring the "perceived" tone timing in line with the assumption that the current inter-tone-interval is similar to previous ones; at the same time, this allows for a decrease in the variance of the estimated inter-tone-intervals, and an increased precision for the prior. The manuscript does indeed state this, as quoted, but I believe the brevity of the current exposition leaves readers in the dark. To further clarify this relationship, the authors could examine the preference for relative vs. absolute timing as a function of the relative precision of the prior (for the Bayesian model with absolute timing prior), as described below (B.4).

C. The authors could also clarify certain points about the Bayesian model, in part through a clearer use of terminology.
    1. There's some general confusion about when the model is dealing with a probability distribution vs. a random number (i.e., a draw from a probability distribution). For example, on page 17, the manuscript currently says, *"the model's representation of this time will be drawn from a gaussian distribution…"*, which I believe is incorrect. Notwithstanding that the precise meaning of the term representation is debatable, to my understanding the model's representation of the tone time ought to *be* the specified gaussian distribution; it is not a *draw* from that distribution, which would suggest something strange like re-sampling from the inferred likelihood distribution to produce a random "observed" time.
    2. Similarly, on page 7, instead of saying, *"We built a model which treats the temporal perception of tones as a gaussian model,"* you might say something like, *"We built a model which represents the timing of each tone as a probability distribution over possible occurrence times. We assume for simplicity that these probability distributions are gaussian…"* The sentence *"Each measurement records the tone's timing with gaussian noise"* should be replaced with something more precise, such as: *"Each tone time is encoded as a gaussian likelihood distribution whose mean is given by the observed timing t_i and whose variance is a constant value \sigma_i = ?, hard-coded into the model."* Where the manuscript says, *"The prior is extrapolated from distributions of previous tones, whose timings and uncertainty are fed to an algorithm to predict the location of the next tone…"* it is not immediately clear whether the algorithm operates on the sequence of previous likelihoods, priors, or posteriors (I assume the latter). This could be cleared up by saying *"The prior is extrapolated from the posterior distributions of previous tones…"*
    3. Contributing to this confusion (again on page 17) is the absence of a clear definition of \mu_i and \mu_s, and inconsistent use of t_i vs \mu_s (which, to my understanding, are the same) – the authors might pick one of these notations and stick with it. It might also be helpful to define a notion of the "perceived" tone timing, something like \hat{t}_i, which I gather would be the mean of the posterior distribution. This would be distinguished from both the "observed" or "sensed" tone timing t_i, which I gather is always the timing of the tone stimulus, and the posterior distribution on tone timing, of which \hat{t}_i would be the mean.
    4. The results about reduced jitter are interesting. They seem to contradict this statement made in the Conclusion on page 9: *"overall performance seems to be the dominating*

feature explaining the timing mechanism preference". Rather, they fit into an overall interpretation that the Bayesian model utilizing an absolute timing prior comes to agree with the relative timing algorithm when there is higher weight on the prior as opposed to the likelihood. I wonder if this overall hypothesis might be tested by quantifying the precision of the prior (relative to both the period and the precision of the likelihood, and perhaps averaged across tones or sampled at the last few tones) in the different stimulus conditions, and showing that, across conditions, it's the relative precision of the prior that dictates whether the absolute Bayesian model is in agreement with the relative or absolute timing algorithms. This could be done for all the previous simulations (e.g., those shown in Fig. 4D-F) to show that the explanatory variable underlying them all is really strength of the prior. This would support other claims made about how the absolute Bayesian model comes to implement the relative timing algorithm for high levels of noise.

D. Finally, a few comments about the oscillatory model and its exposition.
    1. This paragraph on page 5 needs some work to be read clearly: "*We found that when the oscillator was driven by our ambiguous stimuli its phase significantly predicted the position of the last tone according to both algorithms relative and absolute perceptual timing, as long as the mean inter-tone interval of the stimulus maintained a narrow range around the natural frequency of the oscillator. In addition, under these specific conditions (i.e., internal frequency congruent to the stimulus' mean inter-tone interval) we found that manipulating the coupling parameter between the oscillator and the stimulus (see Fig. S1A) modulated the model to better predict early or late according to one or another algorithm (i.e., relative or absolute perceptual timing, see Fig. S1B). Furthermore, by varying the coupling parameter the experimentally found relationship between overall performance and algorithm preference (i.e., better performance leading to absolute-algorithm preference, worse performance to relative) was recovered (see Fig. S1D). Despite these promising results, we found that when the stimulus's mean inter-tone interval varied at the range of the true experiment, no longer matching the natural frequency of the oscillator, its phase prediction power decreased and the relationship between overall performance and algorithm preference vanished (see Fig. S1DE).*"
        - It might be worth changing "predicted" to "was associated with", and noting in particular that it's a *different* phase of the oscillator that is associated with the position of the last tone for each frequency (if I understand correctly). And/or, it might be explained in further depth that, *for each algorithm*, phase concentration at the predicted tone time is being used as a measure of the amount of information present in the oscillator regarding the predicted tone time, or the degree to which the oscillator agrees with that algorithm).
        - The sentence about manipulating the coupling constant is not clear. It might help to make it clear that the coupling constant affects the performance of the model, with performance (as measured by phase concentration at the predicted tone time) increasing as coupling increases from zero and thereafter exhibiting a monotonic relationship to coupling constant, which was different for each algorithm. Please also explain which coupling constant enabled replication of the behavioral results, and how this coupling constant was found. Please also describe the relationship between

this coupling constant and the coupling constant leading to the highest performance for the model – was it the same, or different? If the coupling constant was different, how did the relationship between performance and agreement with absolute vs. relative timing look for the most accurate coupling constant?

- The manuscript states that the relationship between overall performance and algorithm preference vanished with broadband inputs. Is this true only for the same coupling constant that allowed the relationship to be replicated for narrowband inputs? Or did a search for a coupling constant that allowed this relationship to be replicated turn up nothing?

2. It's clear that the term \rho_x adjusts the frequency of the oscillator according to past inputs; it's not clear how each input adjusts the oscillator. Perhaps the authors could mention some of the results on the phase response curve of the Wilson-Cowan oscillator and how those might translate to their model, for those unfamiliar with the model.
3. The authors should cite https://journals.plos.org/ploscompbiol/article?id=10.1371/journal.pcbi.1011154 (*Roman, I. R., Roman, A. S., Kim, J. C., & Large, E. W. (2023). Hebbian learning with elasticity explains how the spontaneous motor tempo affects music performance synchronization. PLOS Computational Biology, 19(6), e1011154*), and give an attempt at comparing their model with the ASHLE model.

Minor/miscellaneous points:

1. In Fig. 1B, unless I'm mistaken, the illustration of relative timing is not correct – tone time should be a monotonic function of time, i.e., a later tone should not occur before an earlier tone. I suggest using a real example here rather than a cartoon.
2. On page 5, I suggest replacing the word "period" with "performance interval" in the following sentence: "The parameter fits confirm a significant period at worse overall performance (when overall performance is less than 8.12) where human behavior significantly prefers relative timing (Fig. 2C)."
3. In the last sentence of the abstract, "priorly" should be "previously".
4. On page 9, there's the phrase "if one reduces the jitter in time between participants"; the "between participants" is a bit confusing here, and I think unnecessary.
5. In Fig. 3A, is the representation of the "sound envelope" accurate? It seems like there's a very long input for each tone - what's the timing there?
6. In Fig. 1C, the "probability of answering late" should be zero or one, I believe. Are the data points jittered in the vertical direction to better show the distribution of points? If so, this should be stated in the legend.
