# Supplementary material for: Adaptive oscillators support Bayesian prediction in temporal processing
Source: PLoS Comput Biol. 2023 Nov 27;19(11):e1011669. doi: 10.1371/journal.pcbi.1011669 (PMC10703266; doi:10.1371/journal.pcbi.1011669)
Supplement: Attachment — Submitted filename: Response to Reviewers.docx [file pcbi.1011669.s015.docx]

**Reviewer #1:**

This paper is an excellent combination of creative experimental work and modeling at multiple levels. It certainly deserves publication in PLOS Comp Bio. My only concerns regard the manner in which the ideas are communicated. There are several points on which clearer communication or some rethinking of underlying ideas would be valuable. There is also a bit of a hole in the fabric of citations holding up the work: previous examples of adaptive oscillators and behavioral models of early/late timing judgement have been largely omitted. Hopefully we can get these minor issues out of the way and get this thing published ASAP.

**We thank the Reviewer for the positive comments about our work and we apologize for the lack of clarity and the missing citations. We very much hope this version attends to all of the Reviewer’s concerns.**

*Main concerns:*

- Throughout, overall participant performance is treated as an independent variable (or, even stranger, as a "mediating variable" between frequency and preferred timing strategy). The authors say that overall performance can "explain" variation in timing algorithm. This choice may make for a clear story, but represents a very strange way of thinking about task performance. The graph relating overall performance with algorithm preference makes sense, but it represents a *relation*, not a function. There must be one or more variables, causally "upstream" of task performance, that parametrize this curve, and that may be affected by frequency. I would love to see the language change throughout to represent this.

**We agree with the reviewer that the previous wording was too specific for our intent and have now sought to clarify this relationship, as just that: a relationship. We have made edits in the Results and Discussion to this effect.**

- Patchy citation coverage: throughout, there is a conspicuous lack of mention of preceding models of period-adjusting oscillators. Jones (and Large, and Palmer, etc.) were already working with this type of model in the late 90's. They were even applied to model interval timing tasks: when the authors discuss using oscillators for "absolute timing" (Absolute vs Relative Timing section), they should mention McAuley, J. D., & Jones, M. R. (2003). Modeling Effects of Rhythmic Context on Perceived Duration: A Comparison of Interval and Entrainment Approaches to Short-Interval Timing. Journal of Experimental Psychology: Human Perception and Performance, 29(6), 1102–1125. <https://doi.org/10.1037/0096-1523.29.6.1102>. Also, Li et al, "For the last time: temporal sensitivity and perceived timing of the final stimulus in an isochronous sequence" does a nice job of reviewing and evaluating possible algorithms for early/late tasks -- definitely worth a read and a citation, and might help to collect some other citations.

**The reviewer is correct, and we apologize for not acknowledging these previous works. We amended this omission in this new version of the manuscript, including the suggested citations in the Introduction as well as in the Discussion section.**

I**ntroduction now reads:**

***“While previous studies have used similar experimental designs (see for example,*** [***(1)***](https://www.zotero.org/google-docs/?alrn1t)***), the computational mechanism driving the subject's responses are often taken for granted and the analyses were conducted according to their assumptions.”***

**Discussion now reads:**

***“AFO contains the oscillatory component which allows for shifting to different models on the basis of performance, and also the adjustment of speed (or period in this case) provided by the architecture of SAM. The combination of the two yields simulations that well resemble human behavior both in terms of behavior across stimulus rates and overall performance, making it a viable candidate for a neural mechanism to underlie temporal prediction. Furthermore, it's important to mention that similar biophysical models have already been used by other authors to explain similar cognitive processes. Specifically, period-adjusting oscillators have been proposed in the music cognition literature as a plausible mechanism underlying beat tracking*** [***(2)***](https://www.zotero.org/google-docs/?QGEPQ3) ***and general metrical structure perception*** [***(3)***](https://www.zotero.org/google-docs/?RK1OYv)***, as well as to explain temporal attention*** [***(4)***](https://www.zotero.org/google-docs/?nJmdGJ)***. Similarly, in a recent study Roman and colleagues show that a non-linear oscillator with Hebbian tempo learning and a pulling force toward the spontaneous individual’s motor frequency is capable of predicting professional musicians’ synchronization on real performance settings*** [***(5)***](https://www.zotero.org/google-docs/?MJ9bDI)***.”***

[**1.	M. S. Li, D. Rhodes, M. D. Luca, For the Last Time: Temporal Sensitivity and Perceived Timing of the Final Stimulus in an Isochronous Sequence. *Timing & Time Perception* 4, 123–146 (2016).**](https://www.zotero.org/google-docs/?YGuvJT)

[**2.	Large, Edward W., Beat tracking with a nonlinear oscillator. *Working Notes of the IJCAI-95 Workshop on Artificial Intelligence and Music* Vol. 24031 (1995).**](https://www.zotero.org/google-docs/?YGuvJT)

[**3.	E. W. LARGE, J. F. KOLEN, Resonance and the Perception of Musical Meter. *Connection Science* 6, 177–208 (1994).**](https://www.zotero.org/google-docs/?YGuvJT)

[**4.	E. W. Large, M. R. Jones, The Dynamics of Attending: How People Track Time-Varying Events. *Psychological Review* 106, 119–159 (1999).**](https://www.zotero.org/google-docs/?YGuvJT)

[**5.	I. R. Roman, A. S. Roman, J. C. Kim, E. W. Large, Hebbian learning with elasticity explains how the spontaneous motor tempo affects music performance synchronization. *PLOS Computational Biology* 19, e1011154 (2023).**](https://www.zotero.org/google-docs/?YGuvJT)

- There are at least three distinct uses of the word "prior" -- the time at which an event is initially expected, the rules (absolute vs. relative) determining the position and spread of that prior, the hard-coded belief that stimuli tend to be rhythmic... This is especially confusing in the first paragraph under "Bayesian Modeling." Phrases like "a prior adjusting timing toward rhythmicity" are confusing since it sounds like the rules determining the priors are changing even though they are not. Overall, some more careful wording around these priors would be helpful to disambiguate between the rules determining the priors, the priors themselves, and the emergent timing algorithms (absolute vs. relative) that act as poles of the space of possible participant strategies.

**We thank the reviewer for this point and agree it is necessary to disambiguate the terminology. We have edited the text so that word prior now should exclusively refer to the distribution of times at which a tone is expected. Such priors are referred to as being based on the absolute or relative timing rules. On the other hand, we now refer to a hard-coded *expectation* of rhythmicity, to leave the term prior exclusively for the expectation of timing in a single event.**

*Fairly minor:*

- This task is a bit unusual in that participants are being asked to make a judgement that does not correspond with a particular reality in the world, only with their own intuitive sense of expectation. Can you offer any reassurance that participants found this task sensical? (Orderly data is a good sanity check, but is there anything else to go on?)

**We agree with the Reviewer on the importance of this question given the lack of a ground truth in the task. While the orderly nature of the data is a sufficient check in our view, we can also emphasize the brief training that participants underwent to familiarize themselves with the task and which we neglected to mention in the previous submission (our apologies!). We feel this brief training also demonstrates that the participants were able to make sense of the task and have included a paragraph on it in the Experimental Design section of the methods and have copy pasted it here:**

***“To familiarize participants with the task, we implemented a brief training period to ensure that the task was clear and sensical. Participants first heard one trial where the tones were perfectly rhythmic, and the probe tone was substantially deviated from the expected time. Then, participants heard 8 more trials where tone timing was jittered but with variance equal to one third of the experimental variance. The probe timing was again significantly deviated such that the correct answer by the two absolute and relative timing expectations would always align. Participants received feedback on each trial of their performance. Finally, the participants were given 5 more trials with timing variance at one half of experimental variance without feedback. After this point, the researcher verified that the participant understood the task and felt clear on their goals before continuing to the main experiment described above.”***

- "As an alternative, we adopt a data driven approach, where the two classical timing computational mechanisms are determined by the participants' responses." Fuzzy language. How about, "where the two classical timing computational mechanisms emerge as opposite poles of participant behavior that ranges from one to the other."

**We thank the Reviewer for suggesting a clearer wording. We corrected the manuscript accordingly.**

- Participants in the 2 Hz condition showed no significant preference, p=0.022. I'm probably missing something, but isn't that "significant"? Are you using a threshold below 0.05?

**We thank the reviewer for catching this error. The p-value is correct, and the text was wrong. We have adjusted to reflect this.**

- "significant period at worse overall performance." "period" is a vague word -- choose something clearer?

**The Reviewer is correct. We modified this sentence to now read:**

***“The parameter fits confirms that for overall low performance (i.e., slope_ABS_ + slope_REL_ < 8.12) human behavior significantly aligns to relative timing (Fig. 2C).”***

- "under certain parameter spaces" -- similarly vague

**We have removed this phrase as it is not necessary. The manuscript now reads:**

***“One of the parameters of the Wilson-Cowan model represents the input coming from other brain regions. Crucially, the value of this parameter modulates the natural frequency of the system.”***

- "Algorithm prevalence" -- for consistency, shouldn't this be "preference"?

**We have chosen instead to refer to “prevalence” or “preference” as “alignment” in keeping with Reviewer 2.**

- "Neural precision" -- was this term explained? Is there a better term for this, or a term that lines up with the math notation? I don't even see where eta is defined.

**Neural precision is the inverse of sensory noise in the model (the standard deviation of the gaussian chosen for the likelihood of each instantiation of the model). The use of eta was a typo it should be 1 / 𝝈. We corrected the Figure accordingly and we also replaced the term Neural by Sensory to make clear this work relies on behavioral data.**

- Fig. 4 F/G -- why not use colors coordinated between F and G?

**We matched colors across panels and modified the caption accordingly.**

- "reduces jitter in time between participants" -- what about this is "between participants"?

**We apologize for this mistake, we deleted “between participants” in the new version of the manuscript.**

- "the range in which relative timing performs best" -- shouldn't this be "the range in which participants seem to be using a more relative timing strategy"?

**We agree and have changed this sentence to read:**

***“As precision in the tone sequence increases, making the sequence closer to isochronous, the function that governs the relationship between overall performance and timing mechanism preference flattens, expanding the range in which predicted participants’ behavior aligns with relative timing.”***

- "...predictions of the model. Showing a reduction..." -- misplaced period.

**Thanks for catching this. We amended it.**

- "Marrian" -- I'm not a fan of this adjectivication of "Marr."

**We modified the sentence to read:**

***“In showing that both the Bayesian and the AFO model can predict features of human behavior, we explain human temporal predictions in sequence at multiple levels of explanation: from Marr’s perspective, computational and algorithmic.”***

- the "d" parameter in the AFO leads to two possible meanings of "dI" -- awkward notation, though I guess it's standard.

**Yes, “d” is the standard notation, but we agree with the Reviewer that it can be interpreted as a derivative. Thus, to avoid confusion, we switched “d” to “g”.**

**Reviewer #2:**

This paper attempts to clarify and reconcile two proposed algorithms for rhythmic timing in humans: absolute timing (in which intervals between events are assumed to be noisy draws from some peaked probability distribution, such as normal) and relative timing (in which the deviations from an assumed underlying rhythm are assumed to be, e.g., normally distributed). In particular, the authors explore the differences between these two timing mechanisms for *irregular* rhythmic stimuli. To do this, the authors use a task in which the goal is to determine whether a “probe tone” is early or late relative to a preceding (quasi-)rhythmic tone sequence. For human subjects, they find that whether subjects seem to rely on absolute or relative timing mechanisms depends on the noisiness of the quasi-rhythmic tone sequence, with absolute and relative mechanisms being preferred at low and high levels of noise, respectively. They show that a similar profile of responses is generated by a modified Wilson-Cowan oscillator which exhibits frequency adaptation, and, interestingly, also by a Bayesian algorithm in which the prior for the timing of the next tone is determined via absolute timing. This paper makes a number of novel and important contributions which potentially bring precision & clarity to the question of absolute vs. relative timing – e.g., exploring rhythmic sequences which exhibit noise or jitter in the timing of successive tones, constructing a version of the Wilson-Cowan oscillator which exhibits frequency adaptation, and demonstrating a preference for relative and absolute timing mechanisms that depends on the level of sensory noise. However, this reviewer feels the paper could go further to clarify the central questions – the relationship between absolute and relative timing and their equivalence, and the relationships between the various models discussed. This would require changes to the exposition (mostly additions) to further elucidate exactly what is happening and exactly what it is that the authors have found. It would be helpful to introduce some terminology to further clarify the differences between the various uses of absolute and relative timing in the manuscript (see A below) and the structure of the Bayesian model (see C below). As far as terminology goes, I have made some suggestions, but anything sufficiently clear, consistent, and appropriate will work. Two possible further analyses would also be useful in this reviewer’s opinion. The first would be to explore which tone sequences lead to different predictions for the absolute and relative timing mechanisms (see B.2 below). The second would be to explore how instantiation of the absolute vs. relative timing mechanism (by the Bayesian model with the prior coming from the absolute timing model) depends on the relative precision of the prior (see C.4 below). I believe these two analyses would greatly improve the manuscript, and it is my guess that they are not too difficult to compute, given the existing analyses, with the second probably easier to execute than the first.

**First, we would like to thank the Reviewer for carefully reading and acknowledging the merits of our study. Secondly, we recognize Reviewers suggestions and integrated them to the best of our ability into this new version of the manuscript.**

Some major points are as follows:

A. As it stands, there is some confusion as to the various uses of absolute and relative timing throughout the paper.

1. The terms “mechanism” and “algorithm” are used with absolute vs. relative timing throughout, but I think the exposition would be helped by further distinguishing at least three different uses of absolute and relative timing in the manuscript. First, absolute and relative timing are two different statistical models of irregular or quasi-regular sequences of events, that lead to different statistics for the intervals between events (see B.3 below).

Then, there are the algorithms that the authors introduce to *predict* the timing of an upcoming event in a quasi-rhythmic sequence based on past events. These are inspired by the absolute and relative timing models, but they are not equivalent. One attempts to impute a period T that minimizes the difference between the observed tone time (t_i) and the tone time expected in the absence of noise (i*T); this corresponds to the relative timing model because it assumes normally distributed differences t_i – i*T. The other attempts to find the most likely inter-event-interval t_{i+1} – t_i, assuming that it will be close to the mean of previous inter-event-intervals; this corresponds to the absolute timing model because it assumes normally distributed inter-event-intervals.

Finally, there is the use of these algorithms to produce priors on unobserved tone times, within a Bayesian framework for tone prediction. This Bayesian framework could also be thought of as an “algorithm”, but it would be helpful to distinguish it from the algorithms described above, perhaps by referring to it exclusively as a “framework” or “Bayesian model” – for example, the “absolute Bayesian model” and the “relative Bayesian model”.

In any case, I think it would be very helpful to outline these distinctions early in the manuscript, perhaps at the beginning of the results section, or perhaps in a “notation” section prior to the results section. It would also be helpful to take pains to specify *which* of these three uses of absolute and relative are intended throughout the manuscript.

**We thank the reviewer for the careful analysis of the absolute and relative distinction. We have added more text at the beginning of the results section to clarify the differences between absolute and relative mechanisms. Clarifying that absolute and relative “mechanisms” refer to perceptual predictions. While we do not overtly distinguish between the perceptual predictions and the method for generating sequences as we only use the relative equivalent and not the absolute, we do clarify early on that the stimuli are generated by the relative method as suggested below. We have also referred to the Bayesian as containing priors driven by either the absolute or relative mechanism. We hope the reviewer agrees that this sufficiently clarifies the different uses of the absolute vs relative distinction, and we thank them for bringing this potential confusion to light.**

2. All three models (human, rhythmic, and Bayesian) are described as showing a “preference” for absolute vs. relative timing, a phrase which I found confusing. In fact, what seems to be happening is that the decisions produced by these systems agree more with the timing determined by regular or absolute timing. I believe terms that reflect this, like “agree with” or “align with” or even “instantiate”, would be easier to understand than “prefer”. In cases where you’re talking about preferential alignment, maybe that phrase could be used.

**We changed the wording along the whole manuscript. We hope this modified version results in a clearer understanding of our work.**

B. The authors ought to include more explanation about the differences between absolute vs. relative timing. There are several places and ways I believe this could be done.

1. The authors should make clear the statistical models/assumptions underlying absolute and relative timing. E.g., unless I’m mistaken, relative timing assumes that $t_{i} = i*T + \epsilon_{i}$, while absolute timing assumes $t_{i} - t_{i-1} = T + \epsilon_{i}$, or that $t_{i} = \sum_{1}^{i} T+\epsilon_{i}=i*T + \sum_{1}^{i} \epsilon_{i}$. Thus, given that noise is assumed to be drawn from the same distribution for both models, the resulting “prediction error” t_i – i*T is differently distributed. E.g., for gaussian $\epsilon_{i}$ , it is assumed to be white noise for relative timing, and brown noise for absolute timing. Thus, as the authors do state in the Methods on page 13, the absolute timing model exhibits a greater probability of drift in the time series. (Other parts of the discussion on page 13 could be brought into the main manuscript, in a section introducing the three uses of absolute & relative.)

Along similar lines, it should be clarified that the time series used in the current experiments are generated from the relative timing model – i.e., they assume a rhythmic event timing T*i and add a gaussian noise to it. This is fine given that the manuscript aims at understanding how humans make *rhythmic* temporal predictions – but it should be mentioned. And, the question of whether humans use different mechanism to estimate the timing of noise that is generated via the absolute timing model could be entertained, although in this reviewers opinion this is unlikely (since the manuscript seems to suggest that humans use an absolute timing-inspired algorithm in either case).

**We thank the reviewer for this analysis. We agree with their mathematical definition of the mechanisms. We have described this difference at the beginning of the results section and to clarify that the relative method was used to generate the sequence.**

2. It should be explained when and how the absolute and relative timing algorithms give different predictions for the timing of the next tone in a sequence. As far as I can tell, the biggest difference here should be that the absolute timing algorithm takes the previous (perceived) tone time at “face value”, adding the average inter-tone-interval to it, while the relative timing algorithm assumes that the previous (perceived) tone time is a noisy observation of some expected rhythmic tone timing, and adjusts the frequency and phase of this underlying/expected rhythm to minimize the sum of the squared differences between t_i and i*T. In other words, for the relative timing algorithm, while some of the noise in tone timing results in a different estimate for T, some it can be “absorbed” by shifting the inferred offsets \eps_i of the “observed” tones from the “beat”. For the absolute timing algorithm, noise in tone timing always contributes directly to changes in the estimated mean and uncertainty of the inter-tone-interval T. In any case, this issue should be clearly unpacked earlier in the manuscript (the current manuscript has something in this direction in the Methods, on page 13).

I would also suggest the authors illustrate one or several tone sequences for which the two algorithms give different predictions. They could replace, e.g., the cartoon example in Fig. 1A (in which actual intervals do not appear to be used) with an actual example where the two algorithms differ in their predictions. To go further, they could isolate the trials in which the two algorithms give very different predictions (e.g., the highest quartile in terms of difference, or predictions that are early for one and late for the other), and see what generalizations can be made about the statistics of the noise on those trials. If the reasoning above is correct, one of the largest contributions here should be the amount of noise in the tone before the probe. An illustrative (accurate, cartoon) example would thus be one in which all tones are exactly rhythmic except the one prior to the probe. The magnitude of the noise in this tone should affect prediction more in the absolute timing algorithm. It’s possible that a sequence in which the inter- tone-intervals are strictly decreasing or increasing would also yield a large difference in the predictions.

**As correctly pointed out by the Reviewer, the more the trial structure departs from perfect isochrony (where the two models predictions are indistinguishable) the more the model predictions depart. Accordingly, if we compute the noise for the last tone (how much it departs from the expected position according to the underlying rhythmic structure), or for the whole sequence of tones, for 500 trials we found a significant correlation between the amount of noise and the difference in the models’ predictions. However, the different models’ categorical answers (i.e., early, or late) not only depend on the trial structure but also on the timing of the probe tone. Even when the two models' predictions significantly depart from each other, if both happen sooner or later than the probe, they will grant the same answer (i.e., early or late). Following the Reviewer's advice, we included a supplementary figure showing these effects and a clarifying couple of sentences in the Results section, now reading:**

***“We generated a series of stimuli fashioned by the relative method as we are interested especially in rhythmic temporal prediction. Crucially, as stated in the previous paragraph, gaussian noise was added to the perfect isochronous structure making the two computational mechanisms of perceptual timing (i.e., absolute and relative) to grant different predictions on when the next tone in the sequence will take place (see Fig. 1AB and Fig. S1).”***

**Figure S1. Trials design and different predictions of the two computational mechanisms of perceptual timing. A.** Relationship between the amount of noise in the last tone of the sequence (how much it departs from the underlying rhythmic structure) and the difference between the probe’s time predicted by the two models. **B.** Relationship between the sum of the noises in the sequence of tones preceding the last one and the difference between the probe’s time predicted by the two models. Panels **A&B** imply that the more the trials depart from the perfect isochronous case, the larger the difference between the models predictions. Additionally, accordingly to how the jitter accumulates on the different models, the noise in the last tone has the strongest effect in differentiating both predictions. Each dot represents one trial and the solid line a linear regression of the data. **C.** Schematic representation of four trials of the 4Hz rhythm condition. Dashed gray lines: Underlying rhythmic structure (i.e., where the tones of the sequence should be presented for a perfect isochronous stimulus). Solid gray lines: Presented tones. Green: Probe tone. Dashed blue and magenta line: Probe’s predicted time according to the relative and absolute perceptual timing, respectively. Congruent trial: both models agree on the probe being early or late. Incongruent trial: there is a disagreement between the models categorical outcome (i.e., probe happens early or late). As exemplified by this figure, the different models’ categorical outcome not only depend on the trail structure but also on the timing of the probe tone. While it’s true that the larger the difference between the models predictions the more likely is to get an incongruent trial and vice versa (i.e., Trials 1 and 2), it's also possible to find trials with: 1. a small difference between models but with the probe taking place in the middle granting an incongruent trial (i.e., early or late, Trial 3); or 2. a large difference between models but both lying on the same side of the probe granting a congruent trial (i.e., early or late, Trial 4). There is no strict functional relationship between the sequence of tones structure and getting a congruent or an incongruent trial.

3. The relationship between Bayesian predictions made using relative and absolute timing – i.e., why it is that the Bayesian framework with the absolute timing prior “looks like” the Bayesian framework with the relative timing prior for high levels of noise – ought to be further explained. The closest the authors come to this is the sentence *“When participants are more uncertain about their own sensory measurements, the expectation of similarity between intervals yields a stronger effect of rhythmicity in the estimation.”* This could be stated much earlier, and the authors could elaborate and clarify the mechanism through which this occurs. I.e., having explained the differences between the two models and algorithms as suggested above, they could state that the precision of the likelihood of each tone plays a crucial role in determining how close that tone’s posterior distribution (and “perceived” timing) is to the prior distribution (which assumes a constant inter-tone-interval, i.e., rhythmicity, for both absolute and rhythmic timing algorithms). Intuitively, when the precision of the observed timing is low, the posterior distribution acts to “adjust” the location of each tone in order to bring the “perceived” tone timing in line with the assumption that the current inter-tone-interval is similar to previous ones; at the same time, this allows for a decrease in the variance of the estimated inter-tone-intervals, and an increased precision for the prior. The manuscript does indeed state this, as quoted, but I believe the brevity of the current exposition leaves readers in the dark. To further clarify this relationship, the authors could examine the preference for relative vs. absolute timing as a function of the relative precision of the prior (for the Bayesian model with absolute timing prior), as described below (B.4).

**We have added a final paragraph to the results clarifying our interpretation that the strength of the prior relative to the likelihood drives the preference towards rhythmicity and further confirmed this using the predictions of Figure 4F. We have also created a supplementary figure explaining the slope difference effect in terms of relative strength of the prior and sensory measurement, which shows that the effect of temporal jitter in the experiment is explained as an increased prior precision relative to sensation. This is fully explained in the added paragraph, copied below, and we believe it very nicely addresses the reviewers suggestion.**

***“While the strength of the prior depends critically on the strength of the sensory measurement (i.e., the likelihood), it is also capped by the uncertainty inherent to the experiment (see Methods and Materials). As such, as the precision of the likelihood increases, the relative dominance of the prior is reduced (See Fig S4B). The prior, even using the algorithm of absolute timing, assumes equivalent intervals over time, i.e., rhythmicity. As such, when the prior has greater dominance (i.e., when sensory measurements have low precision), the outcome is more aligned with the relative mechanism. When the prior has less influence (i.e., when sensory measurements are high and the prior precision is capped), the model is more aligned with the absolute mechanism. This is further confirmed by the predictions of Fig. 4F which shows that when the precision cap of the prior is raised (by reducing temporal jitter), the model is aligned with rhythm for a longer period. We confirmed this hypothesis by explaining the difference in temporal jitters shown in Figure 4F (and Fig. S4A) by explaining the difference in slopes as a function of the relative ratio between Prior Precision and Sensory Precision in the model. We found that the two levels of temporal jitter become aligned from this viewpoint, confirming that the driving feature in alignment to relative or absolute method of timing is driven by the relative dominance of Prior over Sensation, with alignment for the relative mechanism peaking when Prior and Sensory confidence are near equal (Fig. S4C).”***

***Figure S4. Reducing temporal jitter increases relative alignment by strengthening the prior. A.*** *Bayesian simulation of the low jitter (black) and high jitter (magenta) conditions. Equivalent to Fig. 4F.* ***B.*** *Comparison of the Sensory Precision* $\frac{1}{\sigma_{s}}$ *with the Prior Precision* $\frac{1}{\sigma_{p}}$ *in the low jitter (black, solid line) and high jitter (magenta, solid line) conditions. Horizontal dashed lines refer to* $\frac{1}{\sigma}$ *for each experimental jitter:* $\sigma= .1$ *for high jitter (magenta) and* $\sigma= .075$ *for low jitter (black). These are the limit of possible precision of the prior if it were 100% confident where the expected tone location would be (considering the unpredictable variance of the experiment). Gray line dashed line refers to the identity line.* ***C.*** *The low and high jitter conditions are fully aligned when explained by sensory precision relative to the prior* $\frac{\sigma_{p}}{\sigma_{s}}$ *.*

C. The authors could also clarify certain points about the Bayesian model, in part through a clearer use of terminology.

1. There’s some general confusion about when the model is dealing with a probability distribution vs. a random number (i.e., a draw from a probability distribution). For example, on page 17, the manuscript currently says, *“the model’s representation of this time will be drawn from a gaussian distribution...”*, which I believe is incorrect. Notwithstanding that the precise meaning of the term representation is debatable, to my understanding the model’s representation of the tone time ought to *be* the specified gaussian distribution; it is not a *draw* from that distribution, which would suggest something strange like re-sampling from the inferred likelihood distribution to produce a random “observed” time.

**The reviewer is correct that we got the wording wrong in this description. We have adjusted the text to fix this.**

2. Similarly, on page 7, instead of saying, *“We built a model which treats the temporal perception of tones as a gaussian model,”* you might say something like, *“We built a model which represents the timing of each tone as a probability distribution over possible occurrence times. We assume for simplicity that these probability distributions are gaussian...”* The sentence *“Each measurement records the tone’s timing with gaussian noise”* should be replaced with something more precise, such as: *“Each tone time is encoded as a gaussian likelihood distribution whose mean is given by the observed timing t_i and whose variance is a constant value \sigma_i = ?, hard-coded into the model.”* Where the manuscript says, *“The prior is extrapolated from distributions of previous tones, whose timings and uncertainty are fed to an algorithm to predict the location of the next tone...”* it is not immediately clear whether the algorithm operates on the sequence of previous likelihoods, priors, or posteriors (I assume the latter). This could be cleared up by saying *“The prior is extrapolated from the posterior distributions of previous tones...”*

**Thanks to the reviewer for these recommendations to further clarify the model. We have incorporated them directly as requested.**

3. Contributing to this confusion (again on page 17) is the absence of a clear definition of \mu_i and \mu_s, and inconsistent use of t_i vs \mu_s (which, to my understanding, are the same) – the authors might pick one of these notations and stick with it. It might also be helpful to define a notion of the “perceived” tone timing, something like \hat{t}_i, which I gather would be the mean of the posterior distribution. This would be distinguished from both the “observed” or “sensed” tone timing t_i, which I gather is always the timing of the tone stimulus, and the posterior distribution on tone timing, of which \hat{t}_i would be the mean.

**Thanks again to the reviewer on this point. We elected to use** $\mu$ **rather than** $\boldsymbol{t}$ **when referring to the Bayes’ Model, specifically to separate concepts of stimulus generation from how a model would infer that stimulus. In practice,** $\mu_{\boldsymbol{s}}\boldsymbol{=}\boldsymbol{t}_{\boldsymbol{i}}$**. However, conceptually they are different.** $\boldsymbol{t}_{\boldsymbol{i}}$ **refers to the true timing according to stimulus generation,** $\mu_{\boldsymbol{s}}$**to the most probable time of the model infers in sensation when it is driven by a tone at time** $\boldsymbol{t}_{\boldsymbol{i}}$**. In other words, if the model had access directly to** $\boldsymbol{t}_{\boldsymbol{i}}$**, the inference of its location would not be necessary! Still, as the reviewer points out we have not made this distinction entirely hygienically and have now better defined these terms and explained our reasoning. In addition, we have cleaned up the presentation of the equations and more clearly defined all terms in this section.**

4. The results about reduced jitter are interesting. They seem to contradict this statement made in the Conclusion on page 9: *“overall performance seems to be the dominating feature explaining the timing mechanism preference”*. Rather, they fit into an overall interpretation that the Bayesian model utilizing an absolute timing prior comes to agree with the relative timing algorithm when there is higher weight on the prior as opposed to the likelihood. I wonder if this overall hypothesis might be tested by quantifying the precision of the prior (relative to both the period and the precision of the likelihood, and perhaps averaged across tones or sampled at the last few tones) in the different stimulus conditions, and showing that, across conditions, it’s the relative precision of the prior that dictates whether the absolute Bayesian model is in agreement with the relative or absolute timing algorithms. This could be done for all the previous simulations (e.g., those shown in Fig. 4D-F) to show that the explanatory variable underlying them all is really strength of the prior. This would support other claims made about how the absolute Bayesian model comes to implement the relative timing algorithm for high levels of noise.

**We thank the reviewer for this insight and have well incorporated this viewpoint in the paper, particularly in the final paragraph of the results section to clearly identify the role of prior dominance relative to likelihood on perceptual timing. We have also updated the section that the reviewer quotes and make clear that temporal jitter as well is an important influence on the alignment between methods. We clarify that overall performance is a key feature relative to stimulus frequency within a particular temporal variance. Furthermore, we characterize this effect as explained by the Bayesian model as a relative dominance of prior over sensory measurement. We confirm this hypothesis in Supplementary Figure 4, which explains the effect of jitter using the relative strength of the prior to the sensory measurement.**

D. Finally, a few comments about the oscillatory model and its exposition.

1. This paragraph on page 5 needs some work to be read clearly: “*We found that when the oscillator was driven by our ambiguous stimuli its phase significantly predicted the position of the last tone according to both algorithms relative and absolute perceptual timing, as long as the mean inter-tone interval of the stimulus maintained a narrow range around the natural frequency of the oscillator. In addition, under these specific conditions (i.e., internal frequency congruent to the stimulus’ mean inter-tone interval) we found that manipulating the coupling parameter between the oscillator and the stimulus (see Fig. S1A) modulated the model to better predict early or late according to one or another algorithm (i.e., relative or absolute perceptual timing, see Fig. S1B). Furthermore, by varying the coupling parameter the experimentally found relationship between overall performance and algorithm preference (i.e., better performance leading to absolute-algorithm preference, worse performance to relative) was recovered (see Fig. S1D). Despite these promising results, we found that when the stimulus's mean inter- tone interval varied at the range of the true experiment, no longer matching the natural frequency of the oscillator, its phase prediction power decreased and the relationship between overall performance and algorithm preference vanished (see Fig. S1DE).”*

- It might be worth changing “predicted” to “was associated with”, and noting in particular that it’s a *different* phase of the oscillator that is associated with the position of the last tone for each frequency (if I understand correctly). And/or, it might be explained in further depth that, *for each algorithm*, phase concentration at the predicted tone time is being used as a measure of the amount of information present in the oscillator regarding the predicted tone time, or the degree to which the oscillator agrees with that algorithm).

**We have updated the text in the results and methods around Figure S2 to identify Phase Concentration more clearly as containing information regarding the absolute or relative predictions. We thank the reviewer for this suggestion.**

- The sentence about manipulating the coupling constant is not clear. It might help to make it clear that the coupling constant affects the performance of the model, with performance (as measured by phase concentration at the predicted tone time) increasing as coupling increases from zero and thereager exhibiting a monotonic relationship to coupling constant, which was different for each algorithm. Please also explain which coupling constant enabled replication of the behavioral results, and how this coupling constant was found. Please also describe the relationship between this coupling constant and the coupling constant leading to the highest performance for the model – was it the same, or different? If the coupling constant was different, how did the relationship between performance and agreement with absolute vs. relative timing look for the most accurate coupling constant?

**Here, there may be a slight confusion on the part of the reviewer. The analysis of the WC model in figure S2B-F reveals simulations across the range of plausible coupling constants, not for one fixed value. Here we operate under the assumption that individual differences across participants may lead to differences in the coupling parameter and show how these differences lead to a difference in alignment for relative vs absolute timing mechanisms. This is akin to our Bayesian model where we vary the standard deviation of the likelihood across participants to explain the individual differences across groups. We have attempted to clarify this point more readily in the current version.**

- The manuscript states that the relationship between overall performance and algorithm preference vanished with broadband inputs. Is this true only for the same coupling constant that allowed the relationship to be replicated for narrowband inputs? Or did a search for a coupling constant that allowed this relationship to be replicated turn up nothing?

**Again, the panels of Figure S2CE demonstrate the result across all “reasonable” coupling constants. As such, no coupling constants reveal simultaneously a preference for rhythm and absolute. We have attempted to clarify this point in the new text.**

2. It's clear that the term \rho_x adjusts the frequency of the oscillator according to past inputs; it’s not clear how each input adjusts the oscillator. Perhaps the authors could mention some of the results on the phase response curve of the Wilson-Cowan oscillator and how those might translate to their model, for those unfamiliar with the model.

**We thank the author for this suggestion. We have incorporated a panel into the Figure S2 (copied below) on the Wilson Cowan model, demonstrating its phase response curve to a single tone input of 100 ms duration as a function of its coupling parameter. We show that at low coupling levels (those which panel S2B shows significantly more phase concentration for the relative mechanism; color coded blue in the below panel) show small phase adjustments for each input and cross the 0 line (implying synchronization) in two places, one which leads to stable synchronization (near 0 phase), and one which is unstable, with phase responses which push phase difference away from the node (near 3pi/2). With increasing k, the “unstable node” disappears, and phase adjustments increase significantly, with a steeper slope into stable synchronization. These lead to stronger phase resets where phase is most influenced by the most recent tone rather than incorporating small adjustments from all preceding tones. We believe this characteristic is what drives the shift to absolute timing with increasing coupling parameters.**

3. The authors should cite https://journals.plos.org/ploscompbiol/article?id=10.1371/journal.pcbi.1011154 (*Roman, I. R., Roman, A. S., Kim, J. C., & Large, E. W. (2023). Hebbian learning with elasticity explains how the spontaneous motor tempo affects music performance synchronization. PLOS Computational Biology, 19(6), e1011154*), and give an attempt at comparing their model with the ASHLE model.

**We thank the Reviewer for pointing our attention to this relevant paper. We agree that this study aligns with the scope of the current work and the model adopted by them also relies on an oscillator capable of adjusting its internal frequency. Accordingly, we integrated the paper into the discussion. Also, in line with Reviewer’s 1 comments, we apologize for not correctly acknowledging the work done by Ed Large’s team during the past decades using similar models to understand different cognitive processes. To amend these two flows of our previous version the following paragraph has been added to the Discussion:**

***“ AFO contains the oscillatory component which allows for shifting to different models on the basis of performance, and also the adjustment of speed (or period in this case) provided by the architecture of SAM. The combination of the two yields simulations that well resemble human behavior both in terms of behavior across stimulus rates and overall performance, making it a viable candidate for a neural mechanism to underlie temporal prediction. Furthermore, it's important to mention that similar biophysical models have already been used by other authors to explain similar cognitive processes. Specifically, period-adjusting oscillators have been proposed in the music cognition literature as a plausible mechanism underlying beat tracking*** [***(2)***](https://www.zotero.org/google-docs/?3JXQEA) ***and general metrical structure perception*** [***(3)***](https://www.zotero.org/google-docs/?ukIDDc)***, as well as to explain temporal attention*** [***(4)***](https://www.zotero.org/google-docs/?ur05G5)***. Similarly, in a recent study Roman and colleagues show that a non-linear oscillator with Hebbian tempo learning and a pulling force toward the spontaneous individual’s motor frequency is capable of predicting professional musicians’ synchronization on real performance settings*** [***(5)***](https://www.zotero.org/google-docs/?GRAIzM) ***.”***

[**2.	Large, Edward W., Beat tracking with a nonlinear oscillator. *Working Notes of the IJCAI-95 Workshop on Artificial Intelligence and Music* Vol. 24031 (1995).**](https://www.zotero.org/google-docs/?YGuvJT)

[**3.	E. W. LARGE, J. F. KOLEN, Resonance and the Perception of Musical Meter. *Connection Science* 6, 177–208 (1994).**](https://www.zotero.org/google-docs/?YGuvJT)

[**4.	E. W. Large, M. R. Jones, The Dynamics of Attending: How People Track Time-Varying Events. *Psychological Review* 106, 119–159 (1999).**](https://www.zotero.org/google-docs/?YGuvJT)

[**5.	I. R. Roman, A. S. Roman, J. C. Kim, E. W. Large, Hebbian learning with elasticity explains how the spontaneous motor tempo affects music performance synchronization. *PLOS Computational Biology* 19, e1011154 (2023).**](https://www.zotero.org/google-docs/?YGuvJT)

**In the cited study authors explore the motor response of well-trained musicians, accordingly, they included an oscillator for the motor system. The present study is a pure perceptual one with no requirement of two interacting oscillators. For that reason, we did not include simulations with the ASHLE model. However, we believe that an interesting follow up study would be to test the prediction power of the current model (and comparing it with the ASHLE) on a tapping paradigm.**

Minor/miscellaneous points:

1. In Fig. 1B, unless I’m mistaken, the illustration of relative timing is not correct – tone time should be a monotonic function of time, i.e., a later tone should not occur before an earlier tone. I suggest using a real example here rather than a cartoon.

**The reviewer is correct. We corrected Figure 1 accordingly.**

2. On page 5, I suggest replacing the word “period” with “performance interval” in the following sentence: “The parameter fits confirm a significant period at worse overall performance (when overall performance is less than 8.12) where human behavior significantly prefers relative timing (Fig. 2C).”

**We have changed the text to the following to improve clarity:**

***“The parameter fits confirms that for overall low performance (i.e., slope_ABS_ + slope_REL_ < 8.12) human behavior significantly aligns to relative timing (Fig. 2C).”***

3. In the last sentence of the abstract, “priorly” should be “previously”.

**We have made this change.**

4. On page 9, there’s the phrase “if one reduces the jitter in time between participants”; the “between participants” is a bit confusing here, and I think unnecessary.

**We have deleted the unnecessary words**.

5. In Fig. 3A, is the representation of the "sound envelope" accurate? It seems like there's a very long input for each tone - what's the timing there?

**Yes, the envelope is correct. The length of the tone was adjusted differently for each experiment, and it lasted 40% of the mean period. We clarified this by including the following sentence into the methods section:**

***“The tone duration was adjusted for each experiment according to: 0.4 * mean period.”***

6. In Fig. 1C, the “probability of answering late” should be zero or one, I believe. Are the data points jittered in the vertical direction to better show the distribution of points? If so, this should be stated in the legend.

**Yes, a jitter was included to better visualize the data points. The following sentence has been included in the legend:**

***“Dots represent single trial answers with a small vertical jitter added for visualization purposes.”***
